# From Paper to Podium: Quantifying the Translational Potential of Performance Nutrition Research

**DOI:** 10.1007/s40279-018-1005-2

**Published:** 2019-01-22

**Authors:** Graeme L. Close, Andreas M. Kasper, James P. Morton

**Affiliations:** Research Institute for Sport and Exercise Science, Liverpool John Moores University, Liverpool, L3 3AF UK

## Abstract

Sport nutrition is one of the fastest growing and evolving disciplines of sport and exercise science, demonstrated by a 4-fold increase in the number of research papers between 2012 and 2018. Indeed, the scope of contemporary nutrition-related research could range from discovery of novel nutrient-sensitive cell-signalling pathways to the assessment of the effects of sports drinks on exercise performance. For the sport nutrition practitioner, the goal is to translate innovations in research to develop and administer practical interventions that contribute to the delivery of winning performances. Accordingly, step one in the translation of research to practice should always be a well-structured critique of the translational potential of the existing scientific evidence. To this end, we present an operational framework (the “Paper-2-Podium Matrix”) that provides a checklist of criteria for which to prompt the critical evaluation of performance nutrition-related research papers. In considering the (1) research context, (2) participant characteristics, (3) research design, (4) dietary and exercise controls, (5) validity and reliability of exercise performance tests, (6) data analytics, (7) feasibility of application, (8) risk/reward and (9) timing of the intervention, we aimed to provide a time-efficient framework to aid practitioners in their scientific appraisal of research. Ultimately, it is the combination of boldness of reform (i.e. innovations in research) and quality of execution (i.e. ease of administration of practical solutions) that is most likely to deliver the transition from paper to podium.

## Key Points

**Table:** 

The field of sport nutrition has evolved substantially during the last two decades and now encompasses a range of research examining the effects of nutrient availability on modulating cell-signalling pathways through to the more traditional evaluations of ergogenic aids on performance.
The task of translating research to practical interventions to implement in athletic populations has therefore become highly complex, requiring a critical evaluation of the translational potential of the research in question as well as the feasibility of application with specific athletes and sporting domains.
To this end, we present the Paper-to-Podium Matrix, a nine-stage decision-making process to evaluate the translational potential of performance nutrition-related research according to traditional research design indices and feasibility of application.

## Introduction

The emergence of sport nutrition as an accepted academic research discipline can probably trace its roots to the late 1960s with a series of seminal studies examining the role of muscle glycogen and carbohydrate (CHO) availability on exercise capacity and performance [1–4]. In the last 50 years, the field of sports nutrition has grown considerably and the effects of nutrient availability and ergogenic aids on modulating performance, recovery, training adaptations, body composition and immunity are well established [5]. From an academic perspective, there are now multiple scientific journals and international conferences dedicated solely to the dissemination of nutrition-related research, whilst applied sport nutrition research studies continue to be published in mainstream and high-impact physiology journals [6]. From an applied perspective, it is also common practice for sport governing bodies, national institutes, professional sports and Olympic/Paralympic sports to now employ sport nutritionists or dieticians on a full-time or part-time basis.

**Fig. 1:**
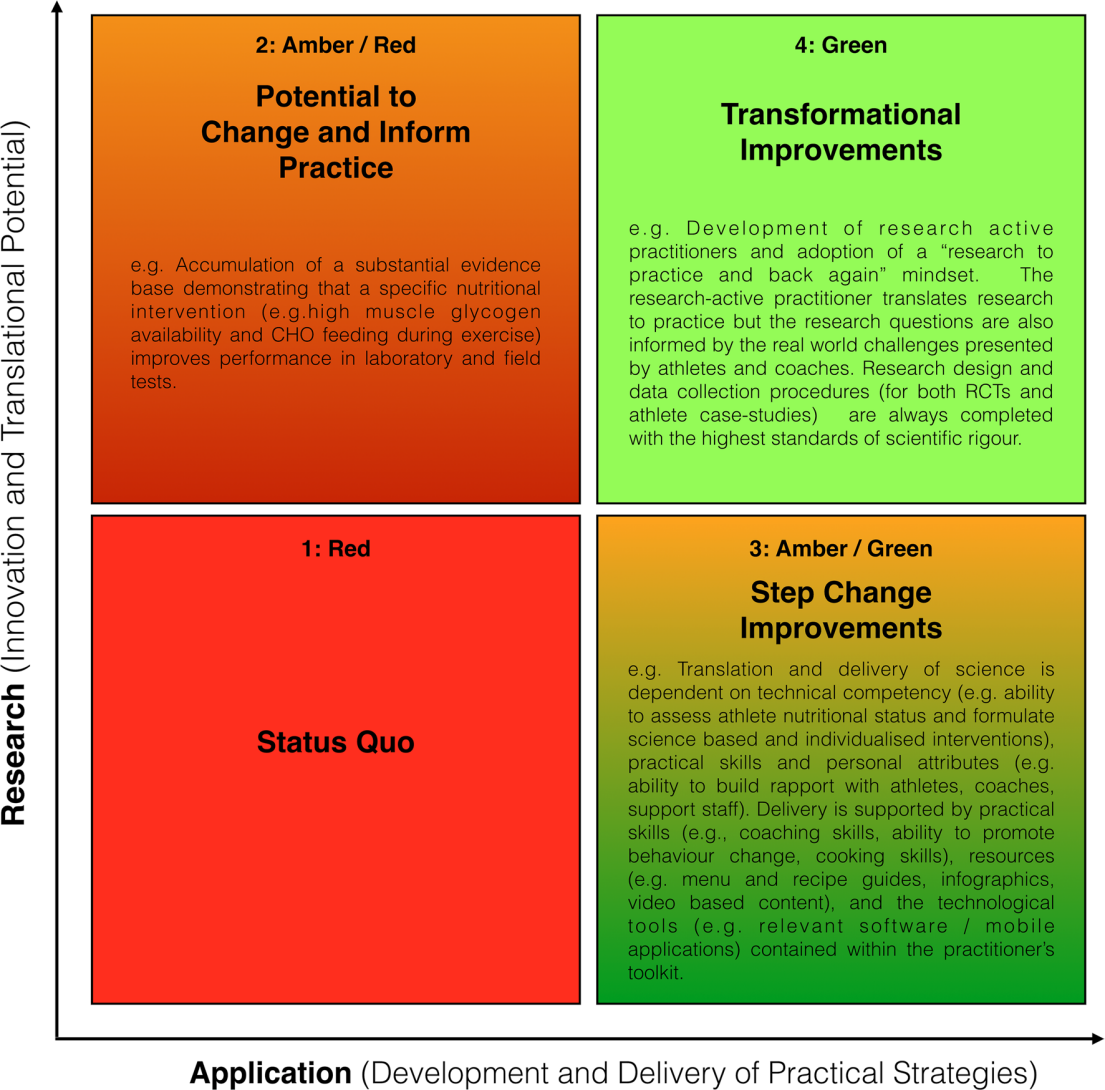
Translation of science to practice: a map of delivery towards improved performance outcomes. In this model, the quality of research is ranked according to the degree of innovation and translational potential whilst practical application is ranked according to the development, delivery and ease of administration of practical strategies. In the absence of developments in research and innovation or alterations to the practical application of the existing science, performance remains as status quo (Quadrant 1). Developments in research and innovation (especially research with translational potential) but without concomitant changes to practical application merely lead to an ‘increased potential’ to deliver improved performance outcomes (Quadrant 2). In contrast, continual improvements in the practical application of existing science are likely to lead to improved performance outcomes (Quadrant 3). Finally, the pursuit of research-informed practice and development of research-active practitioners (who also possess the skill attributes outlined in Quadrant 3) alongside continual improvements in quality of practical application may deliver transformational improvements in performance outcomes (Quadrant 4). *CHO* carbohydrate

As academic researchers and applied practitioners, our current perspectives on practice are based on our experiences of both the laboratory and coalface of elite sport. Whilst our goal is always to ensure the delivery of research-informed practice (see Fig. 1), the conflicting worlds of the “fast” practitioner and “slow” researcher [7] collide on a daily basis. Put simply, elite athletes and coaches who are pursuing the winning margins [8] usually do not have time to wait for the perfect randomised controlled trial, detailed meta-analyses and/or international consensus to reach publication. Despite the well-documented growth of our profession, it could be considered that the field of sport nutrition is one that is highly confusing and contradictory, often fuelled by the growth of social media and infographics that attempt to summarise research findings in 140 characters or in an illustration [9]. There are many potential reasons for such confusion and contradictions that are perhaps unique to the sport nutrition field, including (but not limited to): (1) everybody eats on a daily basis hence the belief that they are all nutrition experts and experiences with food may be cultural and emotional, (2) accurate dietary assessment in free living individuals is highly challenging, and (3) the isolation of single nutrient variables in a study design is nearly impossible. Indeed, we have witnessed numerous examples in our applied practice when research findings have been misconstrued to inform practice. If our field is to continue to grow and truly improve performance, it is our view that step one in the translation of research to practice should always be a well-structured critique of the available scientific evidence, coupled with a decision framework that assesses the potential for application of the scientific evidence in the real-world [10]. To this end, the modern-day sport nutritionist requires expertise in biochemistry, physiology, nutrition and psychology—the latter required to ultimately motivate athletes to change their behaviour.

With this in mind, the aim of the present paper was to provide an operational framework for applied sport nutrition practitioners to critically evaluate the translational potential of research to their chosen sporting arena. Using the “Paper-2-Podium (P-2-P) Matrix” (see Table 1), we provide a checklist of nine criteria with which to evaluate the translational potential of research studies. Like many things in science, there is never a right or wrong answer. Rather, the presentation of our framework and personal reflections is an attempt to provide a protocol to aid practitioners in their scientific appraisal of research and ultimately inform their practice.

**Table 1:** The Paper-2-Podium (P-2-P) Matrix: an operational framework to evaluate the translational potential of performance nutrition research

Score	− 2	− 1	0	+ 1	+ 2
Research context	Non-human cells and no exercise condition (mechanistic study)	Non-human cells but exercise condition (mechanistic study)	Human cell type in vitro study (mechanistic study)	Human participants and exercise performance measures (applied study)	Human participants, exercise performance measures and evaluation of mechanisms (applied and mechanistic study)
Research participants	Levels of participants not reported	Inappropriate age group or training status for the context required	Inappropriate training status of the participants for the context required (with defined criteria) although in required age group	Close to appropriate training status for the context required. e.g. trained level participants when wanting to translate to elite athletes (with defined criteria), and in the required age group	The same training status for the context required, e.g. elite level participants when wanting to translate to elite athletes (with defined criteria) and in required age group
Research design	No control group and no blinding of intervention. No consideration of sample size	Randomisation of participant allocation to treatment in matched pairs design, inclusion of control group but no blinding of intervention. No consideration of sample size	Randomised cross-over trial with repeated measures or matched groups design, inclusion of control group but no blinding of intervention. Sample size commensurate with previous research in the area but no sample size calculations provided	Randomised cross over trial with repeated measures or matched groups design with single blind placebo- controlled conditions. Sample size commensurate with previous research in the area but no sample size calculations provided	Randomised cross over trial with repeated measures or matched groups and double- blind placebo- controlled conditions. A priori sample size calculation provided and justified
Dietary and exercise controls	No reference to dietary or exercise controls	Methods of dietary and exercise control cited (but limited to subject self-reporting) and no objective data provided	Methods of dietary and exercise control cited (but limited to subject self-reporting) supported by relevant objective data	Dietary provision provided by researchers, exercise control cited, supported by relevant objective data but limited replication to real-world context	Dietary provision provided by researchers, exercise control cited, supported by relevant objective data and representative of real-world context
Validity and reliability	No inclusion of familiarisation trial or citation of reliability data and measurement tool error. Exercise protocol not representative of the relevant exercise modality nor valid to real-world context	Inclusion of familiarisation trial but no citation of reliability data or measurement tool error. Exercise protocol not representative of the relevant exercise modality nor valid to real-world context	Inclusion of familiarisation trial and citation of reliability data and measurement tool error. Exercise protocol not representative of the relevant exercise modality nor valid to real-world context	Inclusion of familiarisation trial and citation of reliability data and measurement tool error. Exercise protocol is representative of the relevant exercise modality but limited to a laboratory- based protocol that is not valid to real-world context	Inclusion of familiarisation trial and citation of reliability data and measurement tool error. Exercise protocol is representative of the relevant exercise modality and includes both laboratory- and field- based protocols that are applicable to real-world context
Data analytics	Analytics not reported or performed	Analytics reported but limited to descriptive statistics	Analytics reported and appropriate significance or MBI tests provided	Analytics reported, appropriate significance or MBI tests provided and effect sizes included	Analytics reported, appropriate significance or MBI tests provided, effect sizes included and presentation of individual responses to treatment intervention if appropriate
Having assessed the relevant paper from a research design perspective, the below criteria evaluate the feasibility of application in relation to the practitioner’s chosen sporting context					
Feasibility of application	Outside the budget constraints of the organisation. Complex to implement, e.g. daily long term supplementation and low chance of compliance	Could be within budget constraints but complex to implement and low chance of compliance	Within budget constraints, reasonable to implement and some chance of compliance	Cheap to implement, simple to implement and good chance of compliance	Cheap to implement, extremely simple to implement and minimal risk of non-compliance
Risk/reward	High risk in terms of anti-doping violation or safety of the intervention. No safety data available. Potential to impair performance through high risk of adverse side effects. Optimum dose not stated or unknown	Minimal risk in terms of anti-doping violation but no safety data available. Potential to impair performance through high risk of adverse side effects. Optimum dose not stated or unknown	Minimal risk in terms of anti-doping violation and safety data available. Some potential side effects, e.g. GI discomfort that may reduce performance. Optimal dose suggested but unclear	Minimal risk in terms of anti-doping violation and safety data available. Low risk of side effects that may reduce performance. Optimal dose suggested but unclear	Minimal risk in terms of anti-doping violation and safety data available. Solid evidence of no side effects and optimal dose clear
Timing of intervention	Not age-appropriate. Time available for dosing is not suitable and/or is too close to the major competition to warrant testing the new strategy	Age-appropriate for the athlete. Time available for dosing is not suitable and/or is too close to the major competition to warrant testing the new strategy	Age-appropriate for the athlete. Time available for dosing is not considered optimal but could be effective. Time from the major competition is not sufficient to warrant testing the new strategy	Age-appropriate for the athlete. Time available for dosing is not considered optimal but could be effective. Time from the major competition is sufficient to warrant testing the new strategy.	Age-appropriate for the athlete. Time available for dosing is considered optimal to be effective. Time from the major competition is also sufficient to warrant testing the new strategy
Scores	Negative score Exercise caution when applying the data in practice		0 score—low positive May be an appropriate study to guide implementation although some caution is needed	Moderate to high positive score An appropriate study to guide practice	

Where relevant, the P-2-P Matrix should be used alongside the supporting text in the paper to accommodate the nuances inherent to performance nutrition related research

*GI* gastrointestinal, *MBI* magnitude-based inference

## Research Context

With the emergence of molecular biology in sport nutrition research [11], the scope of contemporary nutrition-related studies can range from cell culture models to whole body physiological responses and outcome measures such as muscle strength or endurance performance [12]. Additionally, the effects of micronutrients [13] and macronutrients [14] are often studied in rodent models that may or may not have application to the exercising human during whole body exercise [13, 15, 16]. For example, the patterns of change in muscle protein turnover have been suggested to be different between humans and rodents [16], and examining the metabolic effects of vitamin C supplements using standard laboratory rodents could also be questioned since such animals (unlike humans) are able to synthesise their own vitamin C [17]. As such, the ecological validity of a particular context does not always translate to the exercising human [18]. Furthermore, whilst identifying suppression or activation of a specific signalling pathway is likely to yield new insights and research questions (that usually arise from studies conducted in non-human cell types), it does not mean that an athlete’s training or nutritional programme should immediately change accordingly. Indeed, changes in messenger RNA (mRNA) in response to an acute exercise and nutritional intervention may not always translate to training-induced changes in cellular protein content and/or enzyme activity, owing to post-transcriptional regulatory processes [19] and becoming accustomed to a given exercise stimulus [20]. In this regard, making the link between acute changes in mRNA and improved exercise performance is a significant leap of faith that often does not occur. As such, it is pertinent to initially consider the cell type under investigation and whether the study is intended as ‘purely’ mechanistic (e.g. elucidating novel molecular mechanisms regulating muscle adaptations) or, alternatively, does the research context immediately lend itself to outcome measures of human performance in the laboratory or field. Initial and careful consideration of the research context therefore provides the platform to further evaluate the translational potential to a specific sporting situation.

## Participant Characteristics

In studies involving human participants, there is often considerable ambiguity in categorising the fitness status and physiological profile of the chosen sample. In this regard, qualitative descriptions such as untrained, recreationally active, trained, well-trained, elite, world-class and professional are regularly cited and often used interchangeably. Such descriptions may or may not be accompanied by detailed physiological profiles and quantification of habitual training loads. In relation to male road cyclists, Jeukendrup et al. provide detailed objective criteria with which to categorise participants as trained, well-trained, elite or world class, an approach that could be considered best practice for endurance-based sports [21]. Alternatively, in team sport scenarios, it may be prudent to simply classify subjects as professional [22–24], semi-professional or amateur on the basis of their monetary income and grading of competition in which they compete. Additionally, youth athletes could be considered elite (when compared with age-matched controls) on the basis of their physical attributes, technical proficiency and/or affiliation to an academy of a professional team [25, 26]. Citation of body composition characteristics (and chosen method of assessment) would also help practitioners to further classify and evaluate the participants under investigation [27, 28]. In the absence of a sport-specific classification system, at the very least, we encourage all researchers to fully describe their participants using as much quantifiable data as possible, such as: age, height, years in sport, years training at an elite level (define what this is), world ranking (if applicable), performance testing (e.g. maximum strength, maximal oxygen uptake [VO_2max_], peak power output, etc.) and body composition, etc. For the applied practitioner, consideration of the participant characteristics is especially important given that the metabolic and physiological responses to exercise are highly dependent on training status [29], thereby affecting the translational potential of nutritional interventions to performance outcome measures. In the context of performance, it is therefore possible that the performance-enhancing effects of a specific intervention (e.g. beetroot juice) could be negated in trained versus less trained participants [30]. As such, the efficacy of any particular nutritional intervention should be investigated in the specific population for which the intervention is intended to be used in practice. In this regard, two excellent examples include recent studies examining the efficacy of ketone diester supplements [31] and ketogenic diets [6] in UCI World Tour cyclists and Olympic race-walkers, respectively.

## Research Design

In the common research question of the effects of a specific nutritional intervention on exercise performance, the ‘gold’ standard research design is often considered as the randomised, counter-balanced repeated measures, crossover design that incorporates a double-blind and placebo-controlled intervention, including sufficient familiarisation trials along with controlling all of the threats to internal validity [32]. For example, when assessing the effects of a novel sports drink on exercise performance, participants must first perform sufficient familiarisation trials, the same participants are tested twice, and the test drink and control drink are taste, colour and flavour matched so both the participants and the researchers are blinded. Assuming appropriate pre-trial dietary and exercise controls (see Sect. 5), a valid and reliable performance test (see Sect. 6) and adoption of suitable statistical procedures (see Sect. 7), this design should allow the researchers to ascertain the true effects of the test drink on performance in the absence of researcher/participant bias and placebo effects [33–35]. Nonetheless, there are many questions in sport nutrition that do not lend themselves to this type of research design. For example, in the case of testing the effects of “real” foods on exercise performance (e.g. high fat vs. high CHO intakes), the research design can lack the double-blind placebo-controlled approach given that both researchers and participants are consciously aware of the food they eat [6]. Similarly, when examining the effects of CHO restriction on training adaptations and performance, a double-blind placebo-controlled design may be lacking [36] unless CHO availability has been manipulated via the provision of taste-, colour- and flavour-matched treatments for adequate subject and researcher blinding [37]. In such scenarios, it is therefore difficult to ascertain the true effects of the dietary intervention given that the participants may be affected by cognitive bias towards any specific dietary approach on performance. Moreover, it is often difficult to fully avoid cognitive bias (i.e. a belief effect) given that some interventions can be obvious (for example the effects of caffeine are hard to mask and most athletes are aware of the performance-enhancing effects of caffeine). In some cases, participants can be matched according to their belief that the intervention will work as another way to control for the lack of a placebo control; for example, only prescribing a low carbohydrate diet to those who believe that this may be an advantage [6]. Even if a placebo is used, it is important that exit interviews are performed, whereby the participants are asked if they have been able to distinguish which group they were in, to help elucidate if the intervention was successful in providing a true placebo control.

In the context of evaluating the effects of common supplements and ergogenic aids (e.g. carnitine, creatine, beta-alanine, vitamin D) on performance-outcome measures and muscle damage, matched-groups design are often more suitable owing to the effects of washout time and/or the repeated bout effect [38–41]. In these situations, ensuring randomised participant allocation to treatment group and matched baseline characteristics (e.g. age, stature, body composition, physiological profile, strength) becomes highly important. Appropriately matched group designs are also important during longitudinal training studies that examine the effects of chronic nutritional interventions on training adaptations (e.g. muscle biochemistry) and performance [6, 42]. Like many things in science, the perfect research design never exists. Nonetheless, practitioners must consider the nuances discussed above prior to making any conclusions on the translational potential of the study in question.

## Dietary and Exercise Controls

Despite published guidelines to standardise dietary intake in nutrition-related performance studies [43], there remains considerable discrepancy amongst researchers. Moreover, there is often confusion between *dietary standardisation* and *dietary replication,* whereby the former involves prescribing a diet for the participant to follow, whilst the latter involves trying to replicate the participant’s regular diet on each visit. Both of these methods have strengths and weaknesses and the choice between them will be dependent upon the overall aim of the trial. Common approaches to *dietary standardisation* may be unsuccessful for a variety of reasons, including adverse physiological effects in response to a standardised diet that are not commensurate with the habitual diet or failure to prescribe diets targeted to deliver specific macronutrient intakes. The actual delivery of *dietary standardisation* procedures may also vary with subjects self-selecting foods based on advice given by the researchers or the researchers administering pre-prepared food packages and/or freshly preparing food at relevant meal times [6, 44], with the latter ensuring the exact foods required are provided. In terms of *dietary replication*, whilst this maintains ecological validity, it often does not control the pre-trial meal between participants. It is crucial that when utilising a *dietary replication* design, researchers verify and report that the participant did actually replicate the diet on subsequent trials. Additionally, reporting and standardisation of exercise in the day(s) prior to the main experimental trial should also be taken into consideration, especially in those situations when failure to do so may lead to differences in pre-exercise muscle glycogen availability, and thereby affect performance.

There are, of course, advantages and disadvantages to many of the common dietary standardisation methods outlined above, including cost and ease of intervention, but also the ecological validity for the research participants. For example, if a specific intervention (e.g. caffeine) is to have a performance-enhancing effect in a particular participant, then perhaps it should be evaluated against the background of the participant’s habitual diet. Alternatively, assessment of the ‘true’ magnitude of effect should perhaps be evaluated in conditions that may be considered best nutritional practice (e.g. CHO loading, pre-exercise meal, additional ergogenic aids, maintaining hydration status, and so on), even if the latter does not conform to the participant’s habitual nutritional practices. The specific issue of fasted or fed trials is particularly challenging given that feeding before and/or during exercise can significantly alter metabolic responses to exercise [45]. As a highlighted example, it is well documented that the effects of exercise on cell signalling [46] or CHO feeding [47] and mouth rinsing [48] on exercise performance are more pronounced when exercise is commenced with low muscle glycogen availability and in the absence of a pre-exercise meal. Nonetheless, these are conditions that are rarely practiced by elite athletes in competition. As another case in point, it is unlikely that elite athletes involved in concurrent training sequences [49] would undertake consecutive aerobic and resistance training sessions in the fasted state or without energy intake between sessions, yet this is an approach that is often used in research studies to evaluate skeletal muscle cell-signalling responses. Finally, the reported effects of specific nutritional compounds on performance (e.g. beetroot juice [50]) and markers of muscle damage, recovery and inflammation (e.g. tart cherry concentrate [51]) are likely to be inflated when researchers administer pre-trial diets that are low in the compound of interest. Taking all these considerations together, we therefore recommend that practitioners carefully evaluate research designs and dietary protocols in relation to the nutritional practices, training loads and training organisational practices that are inherent to their specific sport.

## Validity and Reliability of Exercise Protocols and Performance Tests

One of the most important yet often overlooked criteria for interpreting the translational potential of research is the ecological validity and reliability of the chosen exercise protocol. For example, the one-legged knee extensor model [52] has been used extensively in exercise metabolism research to evaluate local control of muscle metabolism and adaptations to exercise training. From a mechanistic perspective, such a contractile protocol is advantageous given that it provides a within-participant control condition by simultaneously examining responses of the non-contracting contralateral limb [53]. Nonetheless, utilisation of this model to study nutritional interventions is not always applicable to the exercise modalities inherent to sport, owing to the small muscle mass, reduced cardiovascular/thermoregulatory strain and reduced hormonal responses compared with whole body exercise [54]. To this end, it is noteworthy that dose–response studies evaluating the optimal protein dose to stimulate muscle protein synthesis suggest that the absolute protein dose is effectively doubled (i.e. 20–40 g post-exercise protein feeding) when using whole body resistance training protocols [55] versus unilateral exercise protocols [56, 57]. As a related theme, the use of laboratory-based simulations of team sport activity [58–60] is advantageous from a research perspective as they provide a controlled and replicable exercise protocol for studying the effects of dietary interventions, hydration status and ergogenic aids on performance. Nonetheless, given that such protocols lack sport-specific movement patterns (e.g. turning/contacts), it is unsurprising that glycogen utilisation during such protocols [44, 61] is considerably lower than that observed in actual field-based games [62, 63]. In addition to ecological validity, there is also the requirement to carefully consider the reliability of any exercise performance tests as well as the inclusion of any familiarisation trials. Indeed, the issue of familiarisation is especially important given that ‘learning effects’ are more prevalent within untrained populations and with more complex performance tests [64], and therefore authors should report if a learning effect was or was not observed. In this regard, reliability should be established by each laboratory using the same age and training status of participants as those for which the intervention is intended to be implemented. Where possible, reliability should also be quantified across the time-scale of physiological relevance. For example, in the case of assessment of muscle damage and recovery of muscle function, day-to-day reliability of muscle function should initially be quantified over a 7- to 14-day time-scale, and in the absence of any muscle damage and administration of any nutritional or pharmacological intervention [65]. In the context of endurance-type performance tests, it can also be debated as to whether the participant should have access to any internal (e.g. heart rate) or external cues (e.g. power output, running velocity) during testing [66] as well as the validity of time-trial versus exercise capacity tests [67, 68]. For example, in the case of professional road cycling, it could be suggested that the ‘true’ effects of any nutritional intervention or ergogenic aid should always be evaluated with access to external cues given that riders have continual access to power meters, heart-rate data and verbal feedback from accompanying support staff. Additionally, the use of time-trial and exercise capacity tests could both be considered as valid performance measures given that both situations present themselves in the form of designated time-trial stages and the ability to respond to ‘attacks’ on mountain climbs, respectively. In such situations, outcome performance in time trials is heavily dependent on pacing strategies where the ability to respond to ‘attacks’ depends on the psychological and physiological capacity to hold a given power output for as long as possible. Finally, we should remember that the controlled, calm and temperature-controlled laboratory environment is usually never representative of the elite sporting arena. Indeed, it is questionable if the dose–response relationship of caffeine on physical and cognitive performance (without over-stimulation) [69] is still apparent when the athlete has the arousal effects of competing in front of 80,000 spectators. The validity, reliability and real-world context of the chosen exercise protocol should be evaluated in relation to the practitioner’s sport of interest.

## Data Analysis and Presentation

Data analysis is often the most contradictory component of performance nutrition-related research. Indeed, there is often considerable discrepancy in statistical and analytical approaches within and between journals. In the world of applied performance, there has been a recent trend to adopt the approach of magnitude-based inferences [70] as opposed to probability-based testing and the traditional p values, although this approach has been the subject of recent debate in the statistical community [71]. Such an approach is considered to provide a more meaningful interpretation of the potential effect as opposed to conventional statistical significance testing [72]. Nonetheless, in basic science-type studies where the focus is often evaluation of mechanisms, traditional probability testing remains the most common analytical approach. As such, we therefore consider it important that applied practitioners are familiar with the advantages and disadvantages of both methods of choice in order to arrive at an informed evaluation. Additionally, the use of effect sizes can also give a quantitative measure of the strength of the findings [73]. Importantly, researchers should also provide a clear rationale for justification of the chosen sample size (accompanied by power calculations). Finally, the approach to data presentation can also greatly influence how the results are evaluated and interpreted. For example, presentation of group means and standard error (as opposed to standard deviation) does not provide a true representation of the variability of between-subject responses. Rather, researchers often choose to represent variability using standard error (especially in graphical format) for cosmetic reasons [74]. Given that practitioners usually pursue the application of intervention with individual athletes, richer evaluations of data can be made in those instances where researchers present both the magnitude and direction of response within each individual [61]. For example, in a recent study from our laboratory examining the effect of muscle glycogen availability on endurance capacity, we observed that mean exercise capacity was increased by 60 min with high versus moderate pre-exercise glycogen concentration (i.e. 600 vs. 300 mmol kg^−1^ dw) [75]. Nonetheless, the individual magnitude of increase in time to exhaustion ranged from 4 to 113 min. Clearly, evaluation of individual responses can be lost in translation where group means are presented per se.

## Feasibility of Application

Having critically appraised the relevant research according to traditional research design metrics, it is now important to complete the evaluation of the translational potential in relation to the feasibility of application. At this stage, the practitioner is ultimately influenced by the practicalities (and factors limiting delivery) of administering the intervention with a given athlete and within a specific sport. Points to consider (albeit not an exhaustive list) include budget constraints, ease of administration and athlete compliance, risk versus reward (e.g. potential adverse effects on performance, athlete health and potential anti-doping violations) and finally, the time available to test the strategy before peak performance is required. As a simple example related to ergogenic aids, the feasibility of application of pre-competition caffeine ingestion is simpler (and would score higher on the P-2-P Matrix) than that of β-alanine or carnitine supplementation where the cost, compliance and time required to achieve optimal dosing are much greater. Similarly, the risk of potential adverse effects switching to a ketogenic diet in the weeks prior to a major competition are greater than the principles of ensuring adequate CHO intake on the day(s) before competition, as well as during competition itself. When considered this way, it is often the feasibility of application (i.e. the x-axis on Fig. 1) that ultimately determines whether a specific research paper can make the transition from paper to podium.

## Conclusions

Although relatively simple in concept, the translation of research to practice is not always a straight-forward process. Indeed, elite sport is dynamic, unpredictable and often chaotic, none of which can be interpreted by a two-way ANOVA or predicted from the controlled laboratory environment. Despite the continual pursuit and often impatient demand for the latest winning edge, we consider that the starting point for the research-informed practitioner should always be the critical evaluation of the translational potential of the available scientific evidence. Put simply, we must look beyond the abstract, the 140-character tweet and latest infographic in order to truly evaluate the scientific rigour and translational potential of performance nutrition-related research studies. To this end, the development of the P-2-P Matrix (outlined in Table 1) is intended as a simple checklist of criteria to prompt such critical evaluation of research papers. We readily acknowledge that the content and indices of such a framework are not exhaustive. Rather, it was our deliberate aim to provide a time-efficient evaluation tool that can be readily applied by practitioners who all too often operate under the intense time constraints inherent in elite sport. Utilisation of the P-2-P Matrix may help practitioners to personally evaluate a research paper, thereby increasing their own confidence in the intervention they are about to implement, which may ultimately result in a more enthusiastic consultation with the athlete, increasing the chance of an effective intervention. Subsequent to the evaluation of existing research, we also encourage practitioners to conduct field-based research (e.g. case reports or small sample-size studies) with the same degree for scientific rigour and precision of measurement that is requisite for randomised controlled trials. Ultimately, it is the combination of boldness of reform (i.e. innovations in research) and quality of execution (i.e. ease of administration of practical solutions) that is most likely to deliver the transition from paper to podium (Table 2).

**Table 2:** Evaluation of three research papers utilising the Paper-2-Podium Matrix

Criteria	Paper #1	Paper #2	Paper #3
	Kasper et al. [76] Carbohydrate mouth rinse and caffeine improves high-intensity interval running capacity when carbohydrate restricted.	Cobley et al. [77] *N*-Acetylcysteine’s attenuation of fatigue after repeated bouts of intermittent exercise: practical implications for tournament situations	Gomez-Cabrera et al. [78] Effect of xanthine oxidase-generated extracellular superoxide on skeletal muscle force generation
Research context	+ 1 Human participants but no mechanisms tested	+ 1 Human participants but no mechanisms tested	− 1 Rodent muscle given electrical muscle stimulation
Research participants	+ 1 Recreationally active and appropriate age	+ 1 Recreationally active and appropriate age with activity clearly defined	− 2 Rodent study
Research design	+ 1 Randomised, repeated measures double-blind study. Sample size commensurate with previous studies but no sample size calculations provided	+ 2 Between-subjects pair-matched design. Sample size calculated and stated	0 Matched group design although no sample size calculations provided
Dietary and exercise controls	+ 1 Caffeine was restricted for 24–48 h and protein provided prior to sleep low but could be considered limited application to real-world scenario given that true glycogen depletion training protocols are unlikely to be performed prior to bed	− 1 Diet recorded and asked to be repeated but not formally assessed and no objective data	0 All foods provided but not documented and drug administered
Validity and reliability	+ 1 Familiarisation trial cited and reference to reliability statistics. Exercise trial was a laboratory- based protocol consisting of exercise on a motorised treadmill	− 1 Familiarisation trials performed and described; however, no objective reliability data provided. Exercise trial was a laboratory based protocol consisting of shuttle running	− 1 No citation of reliability data of the force measurements and exercise lacking real- world application
Data analytics	+ 1 Analytics reported and individual responses plotted although effect sizes not reported	0 Analytics reported but lacked effect sizes. Lacking individual responses	0 Analytics reported without effect sizes and no individual data
Feasibility of application	+ 1 Cheap to implement and good chance of compliance	0 Cheap to implement but some chance of non-compliance with the loading regime	+ 1 Cheap to implement and good chance of compliance
Risk/reward	+ 1 Minimal risk of anti-doping violation and sufficient safety data available although optimal dose of CHO mouth rinse unknown	− 2 Limited availability of batch- tested product and high risk of side effects that could limit performance. Optimal dosing unknown	-1 Limited availability of batch- tested product, optimal dose unknown, although safety data available
Timing of intervention	+ 2 Age-appropriate and time available for dosing is considered optimal to be effective and time from major competition is sufficient to warrant testing the new strategy.	+ 2 Age- appropriate and time available for dosing is considered optimal to be effective and time from major competition is sufficient to warrant testing the new strategy.	+ 2 Age- appropriate and time available for dosing is considered optimal to be effective and time from major competition is sufficient to warrant testing the new strategy.
Total score/interpretation	+ 10 An appropriate study to guide practice	+ 2 May be an appropriate study to guide implementation, although some caution is needed	− 2 Exercise caution when applying the data into practice

In this scenario the papers were assessed in the context of their translational ability to adult elite athletes. When considering ‘Timing of intervention’ we have assumed that the intervention is age-appropriate, the time available for dosing is considered optimal to be effective, and that the time from major competition is sufficient to warrant testing the new strategy

*CHO* carbohydrate, *MBI* magnitude-based inference
